# Identification of oncolytic vaccinia restriction factors in canine high-grade mammary tumor cells using single-cell transcriptomics

**DOI:** 10.1371/journal.ppat.1008660

**Published:** 2020-10-19

**Authors:** Béatrice Cambien, Kevin Lebrigand, Alberto Baeri, Nicolas Nottet, Catherine Compin, Audrey Lamit, Olivier Ferraris, Christophe N. Peyrefitte, Virginie Magnone, Jérôme Henriques, Laure-Emmanuelle Zaragosi, Sophie Giorgetti-Peraldi, Frédéric Bost, Marine Gautier-Isola, Roger Rezzonico, Pascal Barbry, Robert Barthel, Bernard Mari, Georges Vassaux

**Affiliations:** 1 Université Côte d'Azur, CEA, Laboratoire TIRO, Nice France; 2 Université Côte d'Azur, CNRS, IPMC, FHU-OncoAge, Valbonne, France; 3 Institut de recherche biomédicale des armées, Université de Lyon, Lyon, France; 4 Université Côte d'Azur, INSERM, CNRS, IPMC, Valbonne, France; 5 Université Côte d'Azur, INSERM, C3M, Nice, France; 6 Lucioles Consulting, Chateauneuf-Villevieille, France; Medical University of South Carolina, UNITED STATES

## Abstract

Mammary carcinoma, including triple-negative breast carcinomas (TNBC) are tumor-types for which human and canine pathologies are closely related at the molecular level. The efficacy of an oncolytic vaccinia virus (VV) was compared in low-passage primary carcinoma cells from TNBC versus non-TNBC. Non-TNBC cells were 28 fold more sensitive to VV than TNBC cells in which VV replication is impaired. Single-cell RNA-seq performed on two different TNBC cell samples, infected or not with VV, highlighted three distinct populations: naïve cells, bystander cells, defined as cells exposed to the virus but not infected and infected cells. The transcriptomes of these three populations showed striking variations in the modulation of pathways regulated by cytokines and growth factors. We hypothesized that the pool of genes expressed in the bystander populations was enriched in antiviral genes. Bioinformatic analysis suggested that the reduced activity of the virus was associated with a higher mesenchymal status of the cells. In addition, we demonstrated experimentally that high expression of one gene, DDIT4, is detrimental to VV production. Considering that DDIT4 is associated with a poor prognosis in various cancers including TNBC, our data highlight DDIT4 as a candidate resistance marker for oncolytic poxvirus therapy. This information could be used to design new generations of oncolytic poxviruses. Beyond the field of gene therapy, this study demonstrates that single-cell transcriptomics can be used to identify cellular factors influencing viral replication.

## Author summary

The identification of cellular genes influencing viral replication/propagation has been studied using hypothesis-driven approaches and/or high-throughput RNA interference screens. In the present report, we propose a methodology based on single-cell transcriptomics. We have studied, in the context of oncolytic virotherapy, the susceptibility of different grades of primary low-passage mammary carcinoma cells of canine origin to an oncolytic vaccinia virus (VV). We highlight a fault in replication of VV in cells that originated from high-grade triple-negative breast carcinomas (TNBC). Single-cell RNA-seq performed on TNBC cell samples infected with VV suggested that the reduced activity of the virus was associated with a higher mesenchymal status of the cells. We also demonstrate that high expression of one gene, DDIT4, is detrimental to VV production. Considering that DDIT4 is associated with a poor prognosis in various cancers including TNBC, our data highlight DDIT4 as a candidate resistance marker for oncolytic poxvirus therapy. Beyond the field of cancer gene therapy, we demonstrate here that single-cell transcriptomics increases the arsenal of tools available to identify cellular factors influencing viral replication.

## Introduction

Oncolytic vaccinia virus (VACV) represents a new class of anticancer agent with multiple mechanisms of action. VACV has been shown to act at three distinct levels [1]. VACV infects and selectively replicates in cancer cells, leading to primary oncolysis and resulting in cancer cell destruction [2]. It also disrupts the tumor vasculature [3] and reduces tumor perfusion. Finally, the release of tumor antigens from dead tumor cells participates in the initiation of an immune response that may be effective against tumor cells [1, 4–7]. In humans, VACV, administered intratumorally or systemically has been well tolerated in various clinical trials [4].

Poxviruses are large viruses with cytoplasmic sites of replication and are considered to be less dependent on host cell functions than other DNA viruses. Nevertheless, the existence of cellular proteins capable of inhibiting or enhancing poxvirus replication and spread has been demonstrated. Cellular proteins such as dual specific phosphatase 1 DUSP1 [8] or barrier to autointegration factor (BAF) [9] have been shown to be detrimental to the virus. In contrast, the ubiquitin ligase cullin-3 has been shown to be required for the initiation of viral DNA replication [10]. Furthermore, high-throughput RNA interference screens have suggested the potential role of hundreds of proteins acting as either restricting or promoting factors for poxviruses [10–13]. These studies highlight the importance of cellular factors in VACV replication and spread. Theoretically, over-expression or down-regulation of these putative restricting or promoting factors in carcinoma cells could result in reduced sensitivity and even resistance to VACV when primary oncolysis is considered. The concept of resistance to primary oncolysis by VACV has, so far, not been formally demonstrated. For example, in the field of breast cancer research, *in vitro* testing in established human cell lines and *in vivo* xenografts in mice, has shown clearly and convincingly that VACV has anti-tumor activity against breast cancer [14, 15]. The efficacy of VACV was evident in mouse models of triple-negative high-grade breast carcinoma [15], a pathology associated with poor prognosis and for which new therapeutic options are urgently needed. Nevertheless, these studies were performed using established cancer cell lines that may differ from the actual carcinoma cells present in the tumors.

Spontaneously occurring mammary cancers in dogs are of potential interest in the development of new anticancer agents [16–18] as the classification of canine breast carcinoma is relevant to that of human's [19–23]. Although differences have been highlighted in complex carcinomas [24], simple canine carcinomas faithfully represent human breast carcinomas, both at the histological and molecular level [20, 21]. This is particularly the case for the so called "triple negative carcinomas" (lack of estrogen and progesterone receptors and of epidermal growth factor receptor type 2) [22, 25, 26], for which therapeutic options are currently limited and unsatisfactory.

The aim of the present study was to determine whether differences in VACV -induced ability to kill freshly isolated primary cells from low-grade versus high-grade canine breast carcinomas could be demonstrated. Bulk and single cell RNA-seq were used to analyze events associated with VACV infection and to characterize genes potentially interfering with the VACV cycle.

## Results

### TNBC canine cells show reduced sensitivity to a vaccinia virus-Lister strain deleted in the thymidine kinase gene (VV) compared with TNBC carcinoma cells

**Fig 1:**
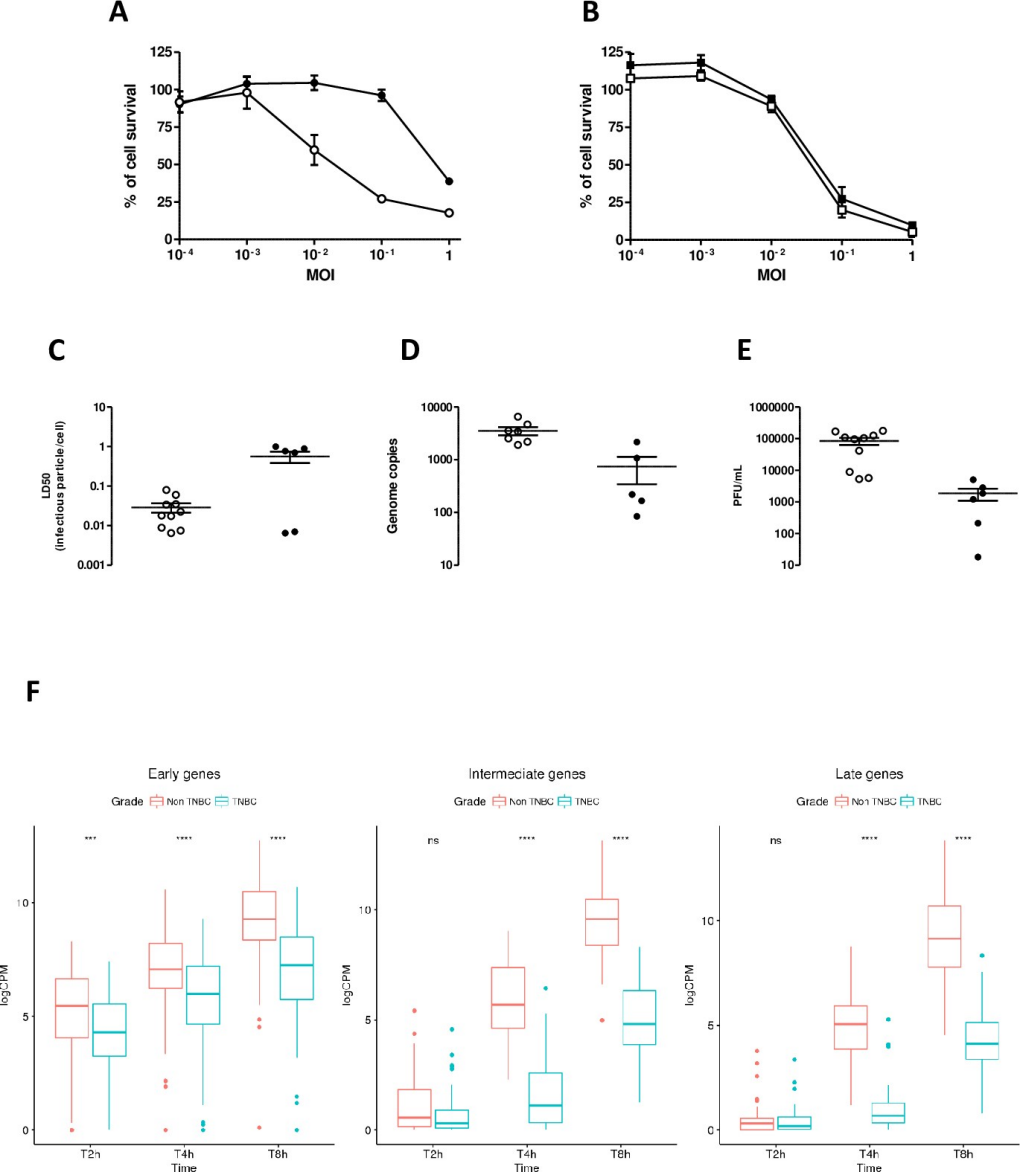
Comparison of the efficacy of VV on non-TNBC or TNBC of different origins. **A.** Primary canine cells (non TNBC: white circles; TNBC black circles) or **B.** human established cell lines (MCF7: white squares, as a non-TNBC established cell line; MDA-MB-231: black squares, as a TNBC established cell line) were infected at different MOIs with VV. Fours days later, the remaining cells were estimated using a MTT assay. The results are presented as a percentage of cell-survival in uninfected cells and are the mean +/- SEM of six different experimental points. The results presented are representative of two independent experiments. **C.** A similar experiment was performed on the cohort of non-TNBC and TNBC cells. For each primary line, two independent experiments involving each six experimental points were performed to calculate the LD50. Each point (white circles: non-TNBC, black circles: TNBC) represents the mean of the two experiments). The bars represent the mean LD50 +/- SE obtained on ten independent non-TNBC and six independent TNBC cultures. LD50 on the Y-axis is expressed as the ratio of infectious viral particles to mammalian cells capable of killing 50% of the cell population. **D.** Twenty-four hours after VV infection (MOI = 0.1), non-TNBC (white circles) or TNBC (black circles) cells were collected, and DNA was isolated and subjected to quantitative PCR to titer the number of viral genomes per well. Each circle represents the mean of two independent experiments performed in triplicate. The mean and SEM of the VV genome copies produced in non-TNBC and TNBC primary cells are presented (**E**). Three days after VV infection (MOI = 0.1), non-TNBC (white circles) or TNBC (black circles) cells were collected, homogenized and the number of infectious particles generated in the culture dishes were titrated. Each circle represents the mean of two independent experiments, each performed in 6 experimental points. The means and SEMs of VV produced in non-TNBC and TNBC primary cells are presented. Details of the samples used to obtain these data (Fig 1A to 1E) are listed in S1 Table. **F.** TNBC (blue) or non-TNBC (red) carcinoma cells were infected at a MOI of 5. At different times post-infection (2, 4, 8 hours), the cells were collected and processed for RNA sequencing. The data represent the levels of early (left panel), intermediate (middle panel) or late (right panel) viral gene expression. CPM on the Y-axis stands for “Counts Per Million”. CPM is the count of sequenced fragments mapping to each relevant gene scaled by the total number of reads times one million. Details of the samples used to obtain these data are listed in S1 Table.

Cells from TNBC or non-TNBC were infected with vaccinia virus-Lister strain deleted in the thymidine kinase gene (VV) at different multiplicities of infection (MOI) and the number of cells remaining in the culture well was monitored after four days. Fig 1A presents an example of dose-response curves showing that non-TNBC cells were more sensitive to VV-mediated cell lysis than TNBC cells. This observation was in sharp contrast to the situation observed in human established cell lines in which MCF7 cells (as a representative of non-TNBC cells) and MDA-MB-231 cells (as a representative of TNBC cells) showed an equivalent sensitivity to VV-induced cell lysis (Fig 1B). The combined LD50 of experiments performed on n = 10 non-TNBC and n = 6 TNBC are presented in Fig 1C and show that non-TNBC cells were 28 fold more sensitive to VV than TNBC cells. By comparison, the LD50 on normal primary canine epithelial cells was around four-fold lower than that of non-TNBC (S1 Fig). Viral production was compared in canine TNBC and non-TNBC cells. Quantitative PCR to determine the number of viral genomes produced on infection (Fig 1D) and titration to determine the number of viral particles showed a reduced number of infectious viral particles produced on infection of TNBC cells (Fig 1E).

### Replication as opposed to viral infection/early stage of viral transcription is affected mainly in canine TNBC cells

We used a vaccinia virus-Copenhagen strain in which GFP expression is driven by an immediate-early vaccinia virus promoter (VACV-Cop-GFP) to infect non-TNBC and TNBC cells. Although the Copenhagen strain is more aggressive that the Lister strain used in the rest of the study, VACV-Cop-GFP represents a very useful tool to visualize and monitor infection/early viral gene transcription. Counting the number of propidium iodide-positive and GFP-positive cells (S2A–S2D Fig) revealed a statistically-significant, 5% difference in infection/early-stage of viral transcription between the two types of cells (S2E Fig). In addition, the difference in mean fluorescence intensity of GFP was statistically non-significant in non-TNBC and TNBC cells (S2F Fig). Propidium iodide staining showed a classical nuclear labelling as well as cytosolic dots (S2B and S2D Fig). These structures are usually found in cells infected with VV and are often referred to as DNA factories or mininuclei [27, 28]. They are cytoplasmic sites of viral DNA replication [27]. The percentage of mininucleus-positive cells in GFP-positive cells eight hours after infection was 53% and 26.6% in non-TNBC and TNBC cells, respectively (S2G Fig). In mininuclei-positive cells, an average of three and one mininuclei were found in the cytosol of non-TNBC and TNBC cells, respectively (S2H Fig). Altogether, these data show that although a difference in the efficacy of infection/early stage of viral transcription can be detected in non-TNC and TNBC cells, the main quantitative difference lies in the number of DNA factories, as in an average population, six-fold more viral DNA factories can be detected in non-TNBC compared to TNBC cells.

Upon infection, early viral genes are rapidly transcribed by the viral RNA-polymerase packaged within the infectious particles [29]. By contrast, the expression of intermediate- and late-viral genes requires *de novo* protein synthesis and viral replication in DNA factories [29]. An implication of the results presented in Fig 1 and S2 Fig is that expression of the early viral genes should be comparable in non-TNBC and TNBC, while expression of intermediate and late genes should be largely impaired in TNBC. To assess this hypothesis, analyses of the kinetics of expression of the early gene E9L and late gene A27L were performed on non-TNBC and TNBC. S3 Fig shows that, the expression of E9L is comparable in non-TNBC and TNBC cells two and four hours after infection and a difference in E9L expression is clearly visible eight hours after infection. The late viral gene A27L was hardly detectable two and four hours after infection and its expression markedly increased eight hours post-infection in non-TNBC, while A27L expression remained low at this time-point in TNBC. To extend these data, the kinetics of bulk viral RNA-expression in non-TNBC versus TNBC cells infected with VV was determined using another pair of donors. Fig 1F shows that the difference in expression of early viral genes in non-TNBC versus TNBC cells is detectable and is statistically significant. However, this difference is much greater for intermediate and late viral gene expression. Altogether, these data suggest that although infection/very early viral gene expression is statistically significantly lower in TNBC than in non-TNBC cells, the number of mininuclei, the replication of the virus and subsequent expression of the intermediate and late viral gene are quantitatively much more affected.

### Single-cell RNA sequencing to dissect VV infection of TNBC cells: impact of the infection on cellular genes

**Fig 2:**
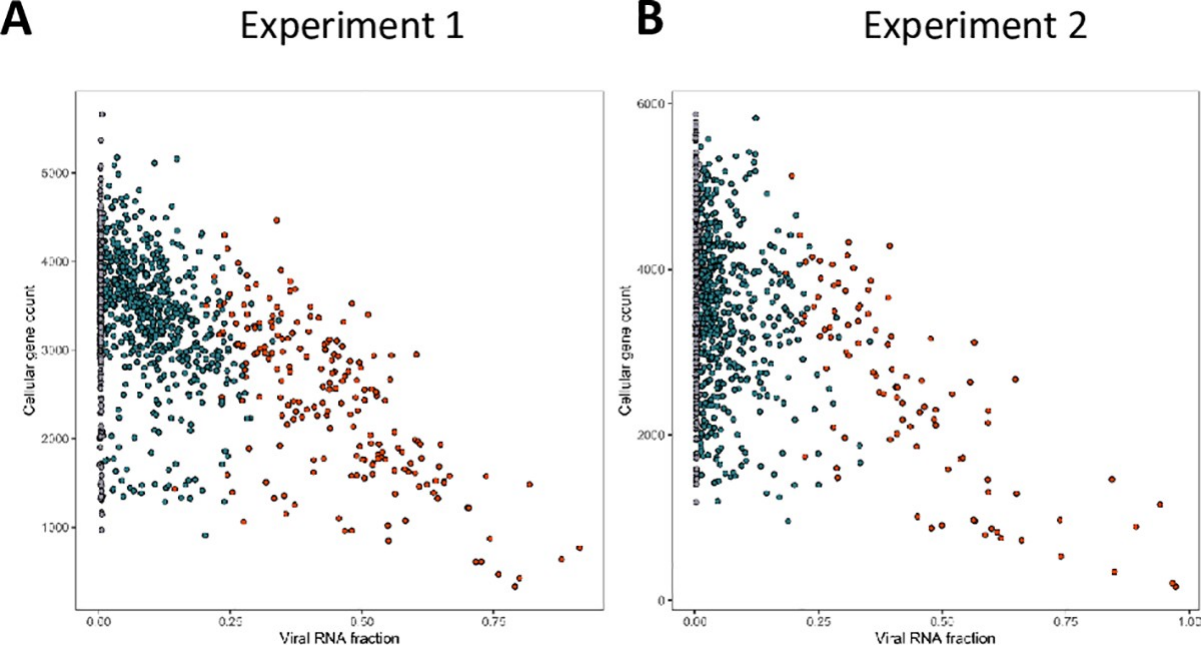
Correlation of cellular and viral genes expression measured by single-cell RNA sequencing. Cells from two independent TNBC were either mock infected or infected with VV (MOI = 5). Six hours later the cells were subjected to the 10X Genomics single-cell protocol, followed by sequencing. Dots represent cells positioned according to the percentage of viral gene expression (x-axis) and the number of cellular genes expressed (y-axis). Black dots represent mock infected cells, blue dots cells expressing early viral genes and red dots cells expressing early and late viral genes. A: experiment 1; B: experiment 2. Details of the samples used to obtain these data are listed in S1 Table.

To characterize further the infection of TNBC cells by VV, we performed single-cell transcriptomic analyses. In these experiments, two independent TNBC primary cell cultures were either mock infected or infected with VV at a MOI of five. Six hours later, the cells were trypsinized and subjected to the 10X Genomics single-cell protocol, followed by sequencing. Fig 2 shows that in the two experiments performed, the number of cellular genes expressed decreased as the extent of viral gene expression increased. More specifically, the number of cellular genes expressed was in the same range in the mock-infected cells (black dots) and in the cells expressing only the early viral genes (blue dots), while the decrease in expression of cellular genes was pronounced in cells expressing late viral genes (red dots).

### Differential expression analysis using naïve, bystander and infected cells

**Fig 3:**
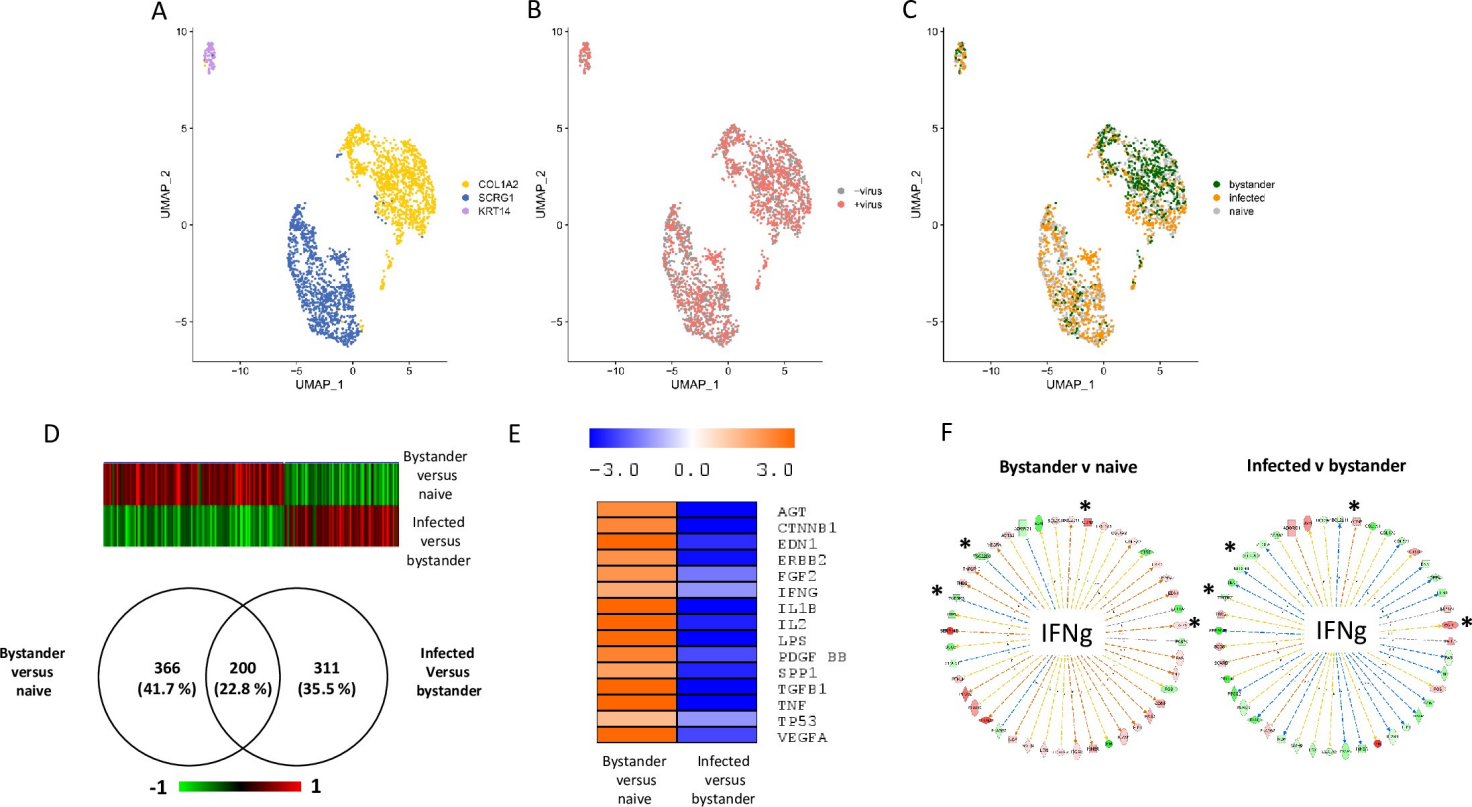
Single-cell transcriptomic analysis of TNBC infected with VV (experiment 1). Cells from TNBC were either mock infected or infected with VV (MOI = 5). Six hours later the cells were subjected to the 10X Genomics single-cell protocol, followed by sequencing. **A**: UMAP representing the three clusters annotated “COL1A2”, “SCRG1” and “KRT14” as these genes are top discriminents for the three clusters. **B**: Repartition of the cells incubated (red) or not (grey) with the virus. **C**: Repartition of naïve, bystander and infected cells in the three clusters. **D**: Venn diagram representing genes that are modulated in bystander versus naïve cells and infected versus bystander cells. The pattern of expression of the genes commonly regulated in the two differential analyses is presented as a heat-map. Red: gene upregulated, green gene down regulated. **E**: Ingenuity Pathways Analysis showing the upstream regulators describing differentially expressed genes in bystander versus naïve cells and infected versus bystander cells. Orange: pathway activated; blue: pathway inhibited. **F**: Example of genes that are part of the IFNγ pathway inversely regulated in bystander versus naïve cells and infected versus bystander cells. Genes in red are up-regulated and genes in green are down-regulated. Only four out of 44 genes (9.1%) are regulated in the same direction in the two conditions. These genes are noted: *. Details of the samples used to obtain these data are listed in S1 Table.

**Fig 4:**
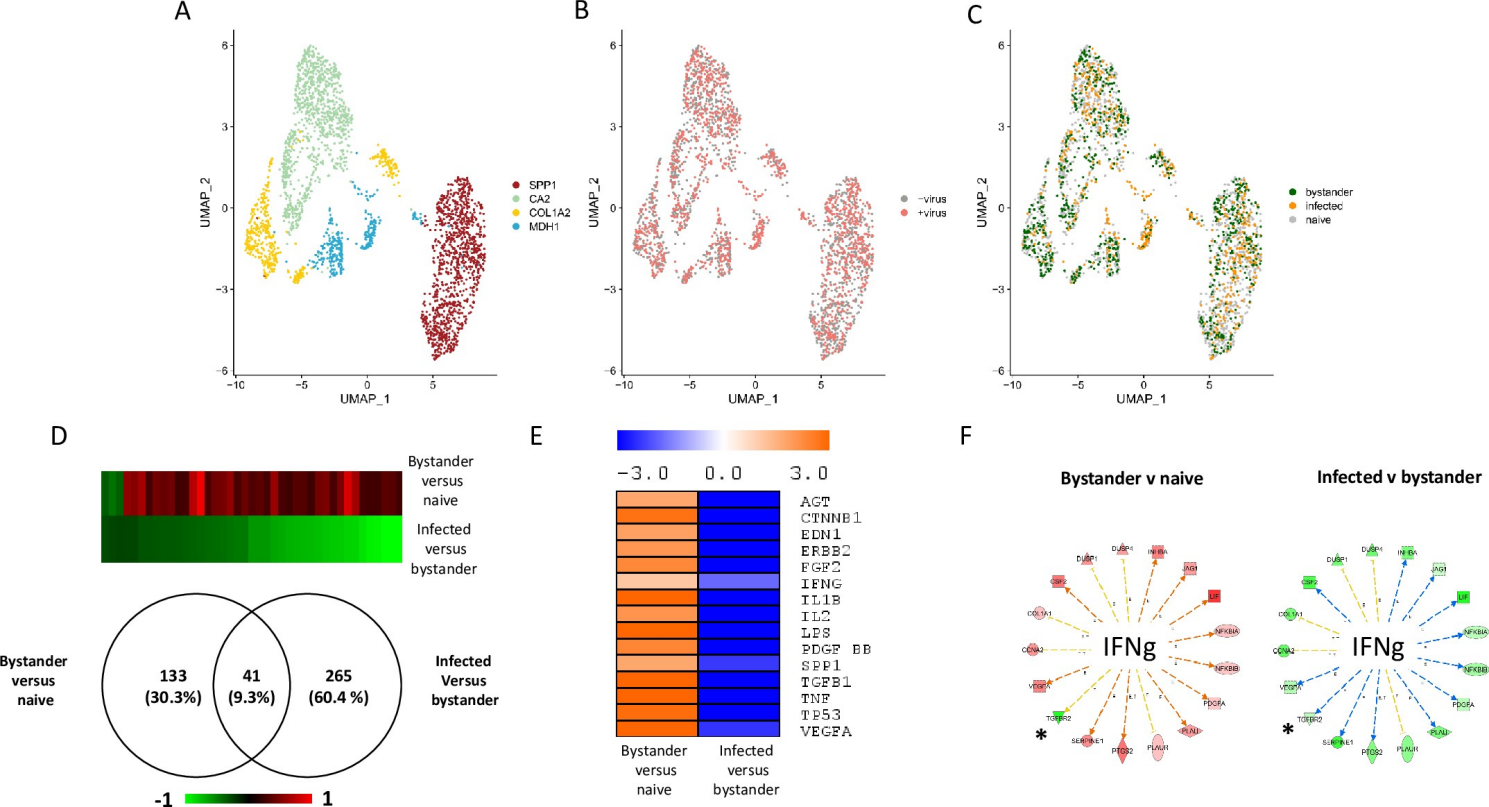
Single-cell transcriptomic analysis of TNBC infected with VV (experiment 2). Cells from TNBC were either mock infected or infected with VV (MOI = 5). Six hours later the cells were subjected to the 10X Genomics single-cell protocol, followed by sequencing. **A**: UMAP plot representing the four clusters annotated “SPP1”, “CA2”, “COL1A2” and “MDH1” as these genes are top discriminant for the four clusters. **B**: Repartition of the cells incubated (red) or not (grey) with the virus. **C**: Repartition of naïve, bystander and infected cells in the four clusters. **D**: Venn diagram representing genes that are modulated in bystander versus naïve cells and infected versus bystander cells. The pattern of expression of the genes commonly regulated in the two differential analyses is presented as a heat-map. Red: gene upregulated, green gene down regulated. **E**: Ingenuity Pathways Analysis showing the upstream regulators describing differentially expressed genes in bystander versus naïve cells and infected versus bystander cells. Orange: pathway activated; blue: pathway inhibited. **F**: Example of genes that are part of the IFNγ pathway inversely regulated in bystander versus naïve cells and infected versus bystander cells. Genes in red are up-regulated and genes in green are down-regulated. Only one out of 17 genes (5.9%) are regulated in the same direction in the two conditions. This genes is noted: *. Details of the samples used to obtain these data are listed in S1 Table.

For the two experiments, a standard statistical analysis using Seurat v3 was performed using cells with a percentage of mitochondrial genes below 25%. On the UMAP plots produced, the cells segregated in three (experiment 1, Fig 3A) and four (experiment 2, Fig 4A) clusters. Each cluster contained both control cells and cells exposed to the virus (Figs 3B and 4B). Three distinct cellular populations were distinguished among the different clusters: naïve cells, defined as cells not exposed to VV; bystander cells, defined as cells exposed to the virus but expressing less than 0.01% of early viral genes; infected cells, defined as expressing more than 0.01% of early viral genes. Naïve, bystander and infected cells were localized onto the UMAP plot (Figs 3C and 4C) and the three cell types were present in all the three (Fig 3C) or four (Fig 4C) clusters. The quantitative analysis of the number of naïve bystander and infected cells in each cluster is presented in Table 1. This analysis indicated that the “COL1A2” clusters showed a higher proportion of bystander cells in the two experiments. Assuming that a higher proportion of bystander cells within a cluster is associated with an increased refractoriness to the virus, we looked at the upstream regulators associated with the two “COL1A2” clusters using Ingenuity Pathway Analysis (IPA) analysis. The transcriptomic signatures of the cells showed, for these two clusters, a pattern highly consistent with “TGF-β” as a major upstream regulator (S4 Fig).

**Table 1:** Repartition of the numbers of naïve, bystander and infected cells in the different clusters. In both cases, the cluster defined as “COL1A2” contains the higher proportion of bystander cells.

Clusters	Naive	Bystander	Infected	Ratio Bystander/Infected
Experiment 1				
COL1A2	510	418	271	1.54
KRT14	42	20	33	0.61
SCRG1	602	75	437	0.17
Experiment 2				
COL1A2	256	156	55	2.84
CA2	503	258	188	1.37
SPP1	576	262	193	1.36
MDH1	136	73	107	0.68

Single-cell transcriptomics provides a unique opportunity to describe, at the molecular level, the effects on cells of both the presence of VV in the culture medium, and of cellular infection with VV. The comparison of the gene expression profiles in bystander versus naïve cells provides information on the effects of the presence of the virus in culture medium on cell that have not been infected, as well as effects that factors secreted by virus-infected cells may have on non-infected cells. The comparison of the gene expression profiles in virus-infected versus bystander cells documents the changes in gene expression triggered by viral infection of cells. The data sets of these two differential expression analyses are presented in S2 Table (experiment 1) and S3 Table (experiment 2). Fig 3D shows that 200 genes were commonly regulated in bystander-versus-naïve and infected-versus-bystander cells. The genes upregulated in the bystander-versus-naïve analysis were found to be downregulated in the infected-versus-bystander analysis and vice-versa (Fig 3D). IPA analysis of the differentially expressed genes provided information on the upstream regulators describing the differences between bystander and naïve cells and between infected and bystander cells. Fig 3E shows that activation of the pathways regulated by, for example, TGF-β1, TNF, IL1β and IFN-γ can be observed when bystander cells are compared with naive cells. This pattern is likely to reflect the reaction of bystander cells to the presence of the virus in the culture medium and to the secretion of various cytokines by cells infected with VV. By contrast, these pathways were inhibited when the IPA analysis was performed on the differentially expressed genes between infected cells and bystander cells (Fig 3E). A similar phenomenon was observed in the second experiment (Fig 4D and 4E). However, in this second experiment, the number of genes modulated in bystander-versus-naïve cells was lower than that observed in experiment 1 (41 genes, see Fig 4D). As the TNBC cells used in experiment 2 are 10 times less sensitive to the virus than those used in experiment 1 (S5 Fig), these differences may be attributed to a blunted ability of cells more resistant to the virus to respond to the presence of the virus in the culture medium and to stimuli secreted by infected cells. Finally, the striking contrasts between bystander-versus-naïve cells and infected-versus-bystander cells were also observed at the level of individual pathways. For example, more than 90% of genes of the IFNγ pathway that were regulated in a particular direction in bystander-versus-naïve cells were regulated in the opposite direction in the infected-versus-bystander cells (Figs 3F and 4F).

### Identification of genes over-represented in bystander versus infected cells

**Fig 5:**
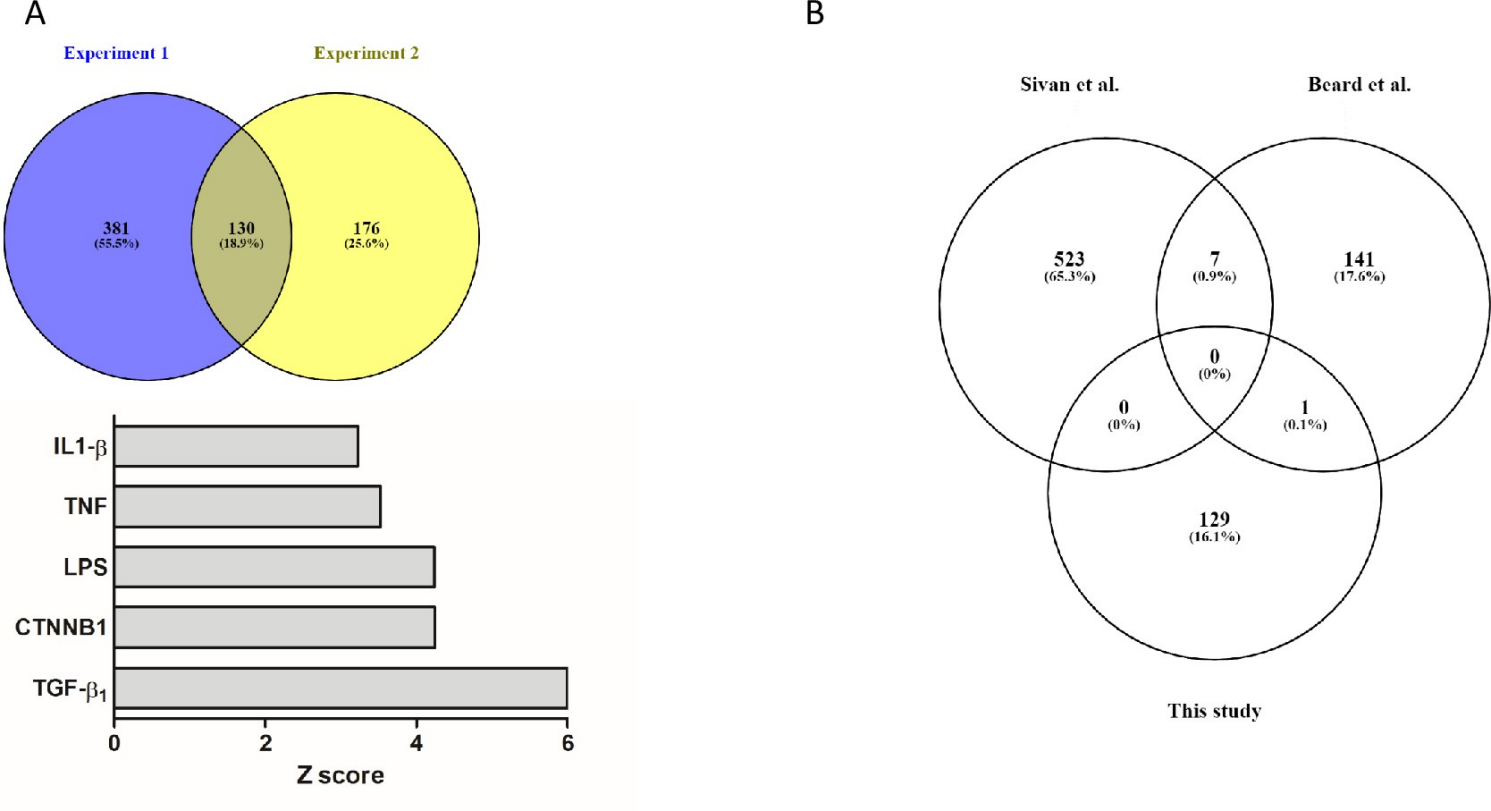
Analysis of the differentially expressed genes in bystander versus infected cells. **A**: Venn diagram of the differentially expressed genes in bystander versus infected cells in experiments 1 and 2. A total of 130 genes are commonly regulated. Ingenuity Pathways Analysis showed that these genes were consistent with an activation of the pathways regulated by TGFβ1, CTNNB1, LPS, TNF and IL1β. The z-score for each pathway is presented. **B**: Comparison of the potential “antiviral genes” in the present study and in the studies of Sivan et al. [11] and Beard et al. [12].

We hypothesized that genes with “antiviral” activities were overrepresented in the bystander compared with the infected population of cells. Fig 5A shows the Venn diagram of the genes differentially expressed in bystander cells in experiments 1 and 2. Under our hypothesis, the 130 genes commonly regulated are candidate genes with antiviral activities (complete list in S4 Table). IPA analysis showed that these genes were consistent with an activation of the pathways regulated by TGFβ1, LPS, TNF, CTNNB1 and IL1β (Fig 5A), suggesting that activation of these pathways is associated with an antiviral action. The comparison of the 130 candidates with genes identified as potential anti-viral genes in high-throughput RNAi screens showed that only one gene, SERPINE1 was found in common with the studies of Beard et al. [12] and none with the study of Sivan et al. [11] (Fig 5B).

### DDIT4 exerts an antiviral activity

**Fig 6:**
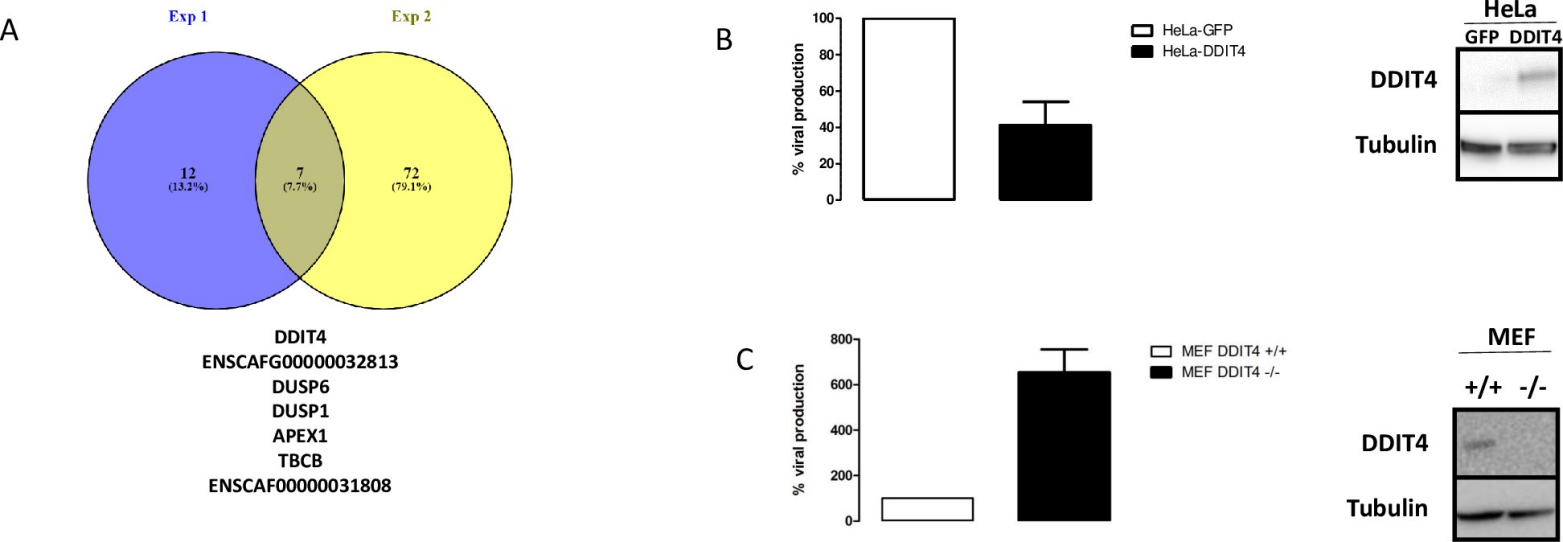
Analysis of the differentially expressed genes in bystander versus infected cells using the “conserved markers” strategy. **A**: Venn diagram of the differentially-expressed genes in bystander versus infected cells in experiments 1 and 2, using a conserved marker analysis. The 7 commonly regulated genes in the two experiments are indicated. ENSCAFG00000032813 and ENSCAF00000031808 are canine genes without human homologs. **B** Three days after VV infection, HeLa-GFP (white bars) or HeLa-DDIT4 (black bars) cells were collected, homogenized and the number of infectious particles generated in 30 μL homogenate were titrated. The results are expressed as the percentage of infectious particles obtained from HeLa-GFP cells. A western blot showing the over-expression of DDIT4 in HeLa-DDIT4 compared to HeLa-GFP is presented. **C**: Three days after VV infection, wild-type MEF (white bars) or MEF DDIT4^-/-^ (black bars) were collected, homogenized and the number of infectious particles generated in 30 μL of homogenates were titrated. The results are expressed as the percentage of infectious particles obtained from wild-type MEFs. A western blot showing the down-regulation of DDIT4 in MEF DDIT4 knock out compared to MEF wild type is presented. For B and C, the results are the mean +/- SD of three independent determinations.

An alternative way to analyze the dataset is to consider each individual cluster in each experiment. This analysis grants less weight to clusters with high numbers of cells. We used the FindConservedMarkers command in Seurat v3 to run differential expression tests cluster by cluster in order to identify the conserved markers between bystander and infected cells. We required a gene to have a log_2_ (Fold Change) > 0.25 and a maximum Bonferroni-corrected P value threshold < 0.05 to be considered as a conserved marker. This analysis identifies genes that are differentially regulated between two conditions (i.e. bystander versus infected) across all clusters in one experiment. We identified 19 and 79 conserved genes in experiments 1 and 2, respectively. Interestingly, only seven genes were conserved between the two experiments (Fig 6A). Two of these genes were canine genes for which human homologs have not been identified (ENSCAFG00000032813 and ENSCAF00000031808). The five remaining genes are APEX1, DDIT4, DUSP6, TBCB and DUSP1. The latter has already been shown to be detrimental to vaccinia virus [8].

We focused on one particular gene (DDIT4) in the list presented in Fig 6A, as high DDIT4 expression has been associated with a poor prognosis in various malignancies including breast cancer [30], and in TNBC [31]. We examined the effect of DDIT4 on VV replication. Infection of HeLa cells overexpressing DDIT4 resulted in a 60% reduction in the production of infectious VV particles compared with control HeLa cells expressing GFP (Fig 6B). Inversely, infection of mouse embryonic fibroblast (MEF) cells obtained from DDIT4 knock out mice resulted in a six-fold increase in the production of infectious VV particles compared with MEF isolated from wild-type mice (Fig 6C).

## Discussion

We demonstrated that oncolysis induced by VV in primary, high-grade canine mammary carcinoma cells was significantly less efficient than in equivalent cells obtained from lower grade tumors. This observation is in sharp contrast with the fact that the same virus was equally efficient in established cell lines from differentiated/low-grade and high-grade human TNBC. Considering the close relationship between the human and canine pathologies [32], it is tempting to attribute this difference in effectiveness of VV to the primary/low passage versus established cell line status of the experimental models. The relevance of established cell lines as experimental systems to develop new cancer therapeutic agents has been questioned in the past and the need for new preclinical models has been highlighted [33]. Patient-derived xenografts have been proposed and are viewed as one of the most relevant model systems in oncology [34]. For a selected number of tumor types including breast cancer, canine tumors recapitulate the features of human tumors and resources from relevant canine tumors have been proposed as tools for the preclinical development of new cancer therapeutics [35]. One of these resources is very-low-passage primary cells grown in serum-free medium. Working with primary low-passage cells has to date been hampered by the small number of cells available from biopsies. It is rare to collect more than 2–3 million carcinoma cells from one biopsy, and, without amplification, this low number of cells considerably restricts the information that can be gathered experimentally. However, with the advent of single-cell transcriptomics, descriptive studies demonstrating whether a therapeutic agent is effective or not can be complemented with high-resolution molecular data.

Single cell transcriptomic has been used previously in the field of infectious diseases. For example, the extreme heterogeneity of influenza virus infection [36] and influenza infection of mouse lungs in vivo [37] have been examined with this tool. However, to our knowledge, this technique has never been applied to the study of poxvirus infection. First, our study confirms the well-documented transcriptional shut-down of cellular genes. In our dataset, this shut-down was correlated to the extent of viral gene expression (Fig 2). A unique feature of single-cell transcritptomic analysis is the possibility to identify different populations of cells that have been in contact with the virus. Cells expressing intermediate and late viral genes express a low number of cellular genes. Their inclusion in the analysis did not provide any particular information for identification of antiviral genes. By contrast, bystander cells (cells exposed to the virus and expressing less than 0.01% of viral genes) provided a unique source of information. Comparison of bystander and naïve cells showed an activation of the pathways regulated by cytokines and growth factors in bystander cells. These activations are likely to be the result of the combined action of the pathogen-associated molecular pattern of the virus as well as autocrine factors secreted by infected and dying cells. The comparison of the activations observed in the two single-cell transcriptomic experiments shows that in the second experiment involving a culture of TNBC cells more refractory to the virus, a blunted response was observed, including the number of genes and the degree of modulation of the regulated genes. Inversely, an inhibition of cytokine and growth factor pathways could be observed when infected versus bystander cells were compared. These inhibitions are likely to have resulted from the expression of viral genes that counteract the cellular responses.

Our study demonstrates that tinformation gathered through single-cell RNA-seq can lead to the identification of pathways with potential antiviral properties. We hypothesized that genes with “antiviral” activities were overrepresented in the bystander compared with infected cell population. Differential expression analysis on the bulk set of data identified 130 genes commonly overrepresented in the bystander populations in the two experiments performed. IPA analysis identified upstream regulators likely to produce these expression profiles (Fig 5A). The constitutive activation of the interferon pathway has been already documented as an “anti-oncolytic” mechanism for different viruses [38, 39], and this pathway, as well as general pathways associated with inflammatory responses (IL1β, lipopolysaccharides (LPS) TNF) were also identified in our dataset with poxvirus infection (Fig 5A). Additional pathways appear to characterize cells from the bystander population: CTNNB1 (β-catenin) and TGF-β (Fig 5A), the latter having already been identified as characteristic for the COL1A2 cluster showing increased “resistance” to poxvirus (Table 1). These two pathways, associated with “inflammatory” pathways, have been widely implicated in the epithelial to mesenchymal transition (EMT) in general and in EMT in breast cancer in particular [40–42]. It is therefore tempting to postulate that cells with a higher “EMT index” would be less sensitive to vaccinia virus. This hypothesis will need to be thoroughly tested in future studies.

A list of 130 candidates is presented in S4 Table. The comparison with genes identified as potential anti-viral genes in high throughput RNAi screens showed that only one gene, SERPINE1, was found in common with these studies (Fig 5B). This low overlap is hardly surprising considering that both the virus and the cells were different in these screens. Nevertheless, this highlights the complexity of the interactions of vaccinia virus with host cells.

An analysis taking into account the individual clusters provided a more stringent test, with only seven genes emerging as commonly over-represented in bystander cells in the two experiments (Fig 6A). Two of those are canine genes with no known human homolog. The five remaining genes are APEX1, DDIT4, DUSP1, DUSP6 and TBCB. The fact that DUSP1 expression has already been demonstrated to be detrimental to vaccinia virus [8] provides a reassurance of the validity of the hypothesis whereby “antiviral” genes are overexpressed in the bystander populations, and highlights the relevance of the methodology we used. The proteins encoded by DUSP1 and DUSP6 are phosphatases with dual specificity for tyrosine and serine. These proteins can dephosphorylate MAPK1/ERK2. DUSP1 has been shown to be involved in the replication and host range of vaccinia virus and in the regulation of host immune responses through the modulation of MAPKs [8]. Although DUSP1 and DUSP6 have been attributed different roles, in immune regulation and development, respectively [43], they may exert antiviral activities through similar mechanisms. Tubulin folding cofactorB (TBCB) has been shown to be required for microtubule network formation [44] but, to our knowledge, an involvement of TBCB in viral infection has never been shown. However, considering the major cytoplasmic reshuffle observed in vaccinia-virus-infected cells, a negative role of TBCB would not be surprising. APEX1 is a major apurinic/apyrimidic (AP) endonuclease in human cells. AP sites are pre-mutagenic lesions and this enzyme is therefore part of the DNA repair machinery [45]. Relationships between APEX1 and viral infection have been documented: inhibition of APEX1 redox activity affects Kaposi’s sarcoma-associated herpes virus [46] and its knock down inhibits HIV1 and HIV2/SIV infection [47]. However, a role of APEX1 in the vaccinia virus lytic cycle has never been reported.

We decided to investigate whether DNA damage-inducible transcript 4 (DDIT4) affects VV replication and we showed, using gain- and loss-of function studies that this is the case (Fig 6B and 6C). DDIT4 is expressed in breast cancer and is associated with a poor prognosis in various cancers including breast cancers [30]. In high-grade triple-negative breast cancers, DDIT4 is also associated with a poor prognosis in human patients [31]. This observation positions DDIT4 as a potential marker that may also be associated with a lower response to oncolytic vaccinia virus. Considering the association of DDIT4 with a worse prognosis in human patients with acute myeloid leukemia, glioblastoma multiform, colon, skin and lung cancers in addition to breast cancer [30], future preclinical and clinical studies should determine the real importance of this gene in the response of various cancer types to oncolytic VV. DDIT4 is an interferon-stimulated gene with anti-retrovial activity [48]. Biochemically, DDIT4 has been described as a negative regulator of the mTOR signaling pathway [49–51]. Rapamycin, a pharmacological inhibitor of the mTOR signaling pathway, has also been reported to reduce virus yield upon VV infection [52]. A possible mechanism may be that mTOR activation results in the phosphorylation of 4E-BP, which in turn releases the translation factor elF4E, the component of el4F that binds to the 5’-cap structure of mRNA and promotes translation [52, 53]. Upon VV infection, the factor elF4E has been reported to be redistributed in cavities present within viral factories [27, 54] where viral translation can proceed. It is therefore tempting to hypothesize that DDIT4, by inhibiting the mTOR signaling pathway, reduces the amount of elF4E available for viral translation. However, considering the complexity of mTOR effects on VV infection [55], the exact nature of the molecular events associated with the inhibitory effect of DDIT4 remains to be elucidated.

Finally, the identification of cellular genes promoting or restricting vaccinia virus infectivity/replication has been studied using hypothesis-driven approaches [8, 9, 56] or high-throughput RNA interference screens [10–13] and in this context single-cell transcriptomics increases the arsenal of experimental tools available. Considering the large transgene capacity of poxviruses, this information could be exploited to generate new generations of oncolytic poxviruses with more efficient direct oncolytic properties.

## Materials and methods

### Cells

Very low passage canine primary cell cultures were provided by Lucioles Consulting. They were derived from a panel of canine primary tissues including normal mammary tissues, hyperplastic lesions, benign tumors, carcinomas in situ and all grades of carcinomas. The list of the different biopsies and their utilization in the different experiments of this report are provided in S1 Table and S5 Table. The tissues were phenotyped using standard histopathology and immunohistochemistry techniques and the ER, PR and HER2 status in relevant samples was determined (S6 Table). Cell survival assays were performed as previously described [57]. BHK21, MCF7, MDA-MB231, HeLa and DDIT4 +/+ and -/- MEF cells were obtained and cultured as previously described [50, 58–62]. Fluorescence imaging and Western blots were performed as previously described [63, 64]. Quantification of the number of fluorescent cells as well as the intensity of fluorescence was performed using the CellQuant program (available at: http://biophytiro.unice.fr/cellQuant/index_html).

### Viruses

A VACV-Lister strain deleted in the thymidine kinase gene (referred to as VV) and VACV-Copenhagen recombinants encoding GFP downstream of a synthetic early promoter (VACV-Cop21 and VACV-Cop 32) were described previously [65, 66]. Vaccinia virus titration was performed on BHK21 cell monolayers infected for two days and stained with neutral red. Lentiviruses (encoding either GFP or DDIT4) were purchased from Sigma-Aldrich and are part of the MISSION TRC3 LentiORF collection.

### qPCR assay for generic detection of Orthopoxvirus

DNA was extracted using the QIAamp DNA Mini Blood kit (QIAgen). The qPCR assay used for the detection of orthopoxviruses was a modification of the assay described by Scaramozzino et al [67]. Probes were designed to amplify a 157 bp fragment of orthopoxvirus A27L gene. Each qPCR assay was carried out in 20μl reaction mixture containing 5μl extracted DNA as template, 400 nM of each primer and 250 nM of probe and 10 μl IQ Supermix for QPCR (Biorad). The reaction was performed as follows: 1 cycle at 95°C for 3 min, followed by 45 cycles each at 95°C for 15 s, followed by 62°C for 60 s. A fluorescence reading was taken at the end of each 62°C step. Data acquisition and analysis were carried out with the Bio-Rad CFX Manager software 3.1. Sample curves were analyzed using the second derivative. Each DNA solution was assayed in duplicate per qPCR assay. Standard curves were generated from serial dilution of a solution of pVACV_Lis-A27L enabling absolute quantification.

### qPCR assay for generic detection of early and late genes, E9L and A27L respectively, and a housekeeping gene

Total RNA was extracted using the RNAeasy kit (Qiagen). Reverse-transcription was performed using the PrimeScript RT Reagent Kit (Perfect Real Time, TAKARA). The A27L (late gene) qPCR assay was performed as described above. The E9L (early gene) and beta-actin qPCR were performed as described by Kulesh et al.[68] and Piorkowski et al. [69], respectively, with slight modifications. Each qPCR assay was carried out in 20μl reaction mixture containing 5μl cDNA as template, 300 nM of each primer and 100 nM of probe and 10 μl IQ Supermix for qPCR (Biorad). Each DNA solution was assayed in duplicate per qPCR assay. Standard curves were generated from serial dilution of a supernatant of a vaccinia virus-Copenhagen strain-infected culture. PCR efficiencies of both the targets genes and the reference gene were between 90% and 110% and did not differ by more than 10%. The delta Ct method was then used for relative quantification.

### Bulk RNA sequencing

Cells were either mock infected or infected at a MOI of 5 with VV. At different time after infection, cells were washed with PBS. Poly(A) RNAs were purified using a Dynabeads mRNA purification kit (Invitrogen) and fragmented for 7 min at 95°C. Libraries were then generated with the Ion Total RNA seq kit V2 (Life technologies) and sequenced on the Ion Proton system with P1 chip V3 following the manufacturer’s instructions. Reads were aligned to the dog genome release canFam3 and the Vaccinia virus genome release NC006998 with bowtie v2-2.2.4. Quantification of genes was then performed using HTSeq-count release HTSeq-0.6.1 with “—minaqual = 0—mod = intersection-nonempty” options. To assess the response differences to the viral infection between non TNBC and TNBC samples, we used the classification of early, intermediate and late poxvirus genes described previously [70]. P-values on boxplots were calculated by the Wilcoxon rank sum test.

### Single cell RNA-sequencing

Single cell suspensions were converted to barcoded scRNA-seq libraries using the Chromium Single Cell 3’ Library, Gel Bead & Multiplex Kit and Chip Kit (10x Genomics), aiming for an estimated 2000 cells per library and following the manufacturer’s instructions. Samples were processed using kits pertaining to V2 barcoding chemistry of 10x Genomics. Libraries were sequenced on an Illumina NextSeq500 with a High Output v2 kit (150 cycles): the forward read had a length of 26 bases that included the cell barcode and the UMI; the reverse read had a length of 98 bases that contained the cDNA insert. Raw sequencing FASTQ files were analyzed within 10x Genomics CellRanger suite (v1.3.0) with a transcriptome reference composed of canFam3 Canis familiaris genome build and the Vaccinia virus complete genome (NCBI reference sequence NC_006998).

### Single cell gene expression quantification and determination of the major cell types

Raw gene expression matrices generated with 10xGenomics CellRanger suite (v1.3.0) were loaded and processed into R (version 3.5.2). Both experiments were analyzed independently using the Seurat R package (version 3.0.0). First, all cells that had over 25% of mitochondrial RNAs were removed. From the remaining cells, gene expression matrices were normalized using the SCTransform method. To reduce dimensionality, variably expressed genes were summarized by principle component analysis, and the 30 PCs were further summarized using UMAP dimensionality reduction. Both samples (i.e. infected and not infected) from the two experiments (i.e. 1 and 2) were then aggregated using FindIntegrationAnchors and IntegrateData functions preceded by the PrepSCTIntegration function described in the Seurat development version. Clusters were called using a low resolution of 0.1, and gene markers were assessed using FindAllMarkers function with standard parameters.

## Supporting information

S1 Fig Efficacy of VV on primary normal canine mammary epithelial cells. Primary normal canine mammary epithelial cells cells were infected at different MOIs with VV. Fours days later, the remaining cells were estimated using a MTT assay. The results are presented as a percentage of cell-survival in uninfected cells and are mean +/- SEM of six different experimental points. This result is representative of two independent determinations.
(PPTX)

S2 Fig Primary canine TNBC cells infected with a vaccinia virus-Copenhagen exhibit a reduced numbers of mininuclei compared to primary canine non-TNBC cells. Non-TNBC (A, B) or TNBC (C, D) cells were infected with a vaccinia virus-Copenhagen strain recombinant in which GFP expression is driven by an immediate-early vaccinia virus promoter (MOI = 5). Three hours after infection, the cells were fixed and stained with propidium iodide (PI). A and B: GFP staining; C and D: propidium iodide staining; M: mininuclei. **F, G, H, I.**Non-TNBC (white bars) or TNBC (black bars) cells were infected with a vaccinia virus-Copenhagen strain recombinant in which GFP expression is driven by an immediate-early vaccinia virus promoter (MOI = 5). Three hours after infection, the cells were fixed and stained with propidium iodide (PI). The number of PI and GFP positive cells was determined. The percentage of GFP+ cells (**F**), the mean GFP fluorescence per cell (G), the percentage of mini-nuclei in GFP+ cells (**H**) and the number of mini-nuclei in nuclei-positive cells (**I**) are presented. (*** p < 0.001; ** p < 0.01; * p < 0.05; n.s: p > 0.05). The data were obtained from the analysis of 120 images obtained from 2 non-TNBC and 2 TNBC from 4 primary canine specimen. The detail of the samples used to obtain these data is listed in S5 Table.
(PPTX)

S3 Fig Differential expression of the E7L and late A27L genes in a pair of non-TNBC and TNBC canine primary cells. Non-TNBC (white bars) or TNBC (black bars) cells were infected at MOI of 0.1 and total RNA were extracted 2, 4 and 8 hours post-infection. The levels of expression of E9L and A27L were determined by quantitative RT-PCR and normalized to the expression of beta-actin.
(PPTX)

S4 Fig Network of genes associated with an activation of TGF-b1 in the clusters COL1A2. The genes caracteristic of the clusters COL1A2 in the two experiments were anaylsed using IPA. In both cases the transcriptomic signature is associated with TGF-b1 and the top upstream regulator. **A**: Experiment 1: 69 out of 99 genes consistent with an activation of TGF-b1 (z score 7,037). **B**: Experiment 2: 77 out of 102 genes have measurements consistent with an activation of TGF-b1 (z score 7,933).
(PPTX)

S5 Fig Comparison of the efficacy of VV on cells from the two TNBC samples used in the single cell transcriptomics experiment. Primary canine cells from TNBC origin used in single-cell transcriptomics experiments 1 and 2 were infected at different MOIs with VV. Fours days later, the remaining cells were estimated using a MTT assay. The results are presented as a percentage of cell-survival in uninfected cells and are mean +/- SEM of six different experimental points. This result is representative of two independent determinations.
(PPTX)

S1 Table S1A Table: Differential expression of genes in bystander versus naïve cells (Experiment 1). S1B Table: Differential expression of genes in infected versus bystander cells (Experiment 1).
(DOCX)

S2 Table S2A Table: Differential expression of genes in bystander versus naïve cells (Experiment 2). S2B Table: Differential expression of genes in infected versus bystander cells (Experiment 2).
(XLSX)

S3 Table List of genes 130 genes commonly regulated in bystander versus infected cells in the two experiments. (XLSX)

S4 Table Breed, age and type of tissue used to extract low passage, primary cells. (XLSX)

S5 Table Histological types and utilization of the cells from the different specimen in the figures of the manuscript. (DOCX)

S6 Table Estrogen receptor (ER), progesterone receptor (ER) and human epidermal growth factor receptor 2 (HER2) status of relevant biopsies used in this study. (DOCX)
