# Supplementary material for: Identification of oncolytic vaccinia restriction factors in canine high-grade mammary tumor cells using single-cell transcriptomics
Source: PLoS Pathog. 2020 Oct 19;16(10):e1008660. doi: 10.1371/journal.ppat.1008660 (PMC7595618; doi:10.1371/journal.ppat.1008660)
Supplement: S1 Fig — Primary normal canine mammary epithelial cells cells were infected at different MOIs with VV. Fours days later, the remaining cells were estimated using a MTT assay. The results are presented as a percentage of cell-survival in uninfected cells and are mean +/- SEM of six different experimental points. This result is representative of two independent determinations. (PPTX) [file ppat.1008660.s001.pptx]

## Slide 1
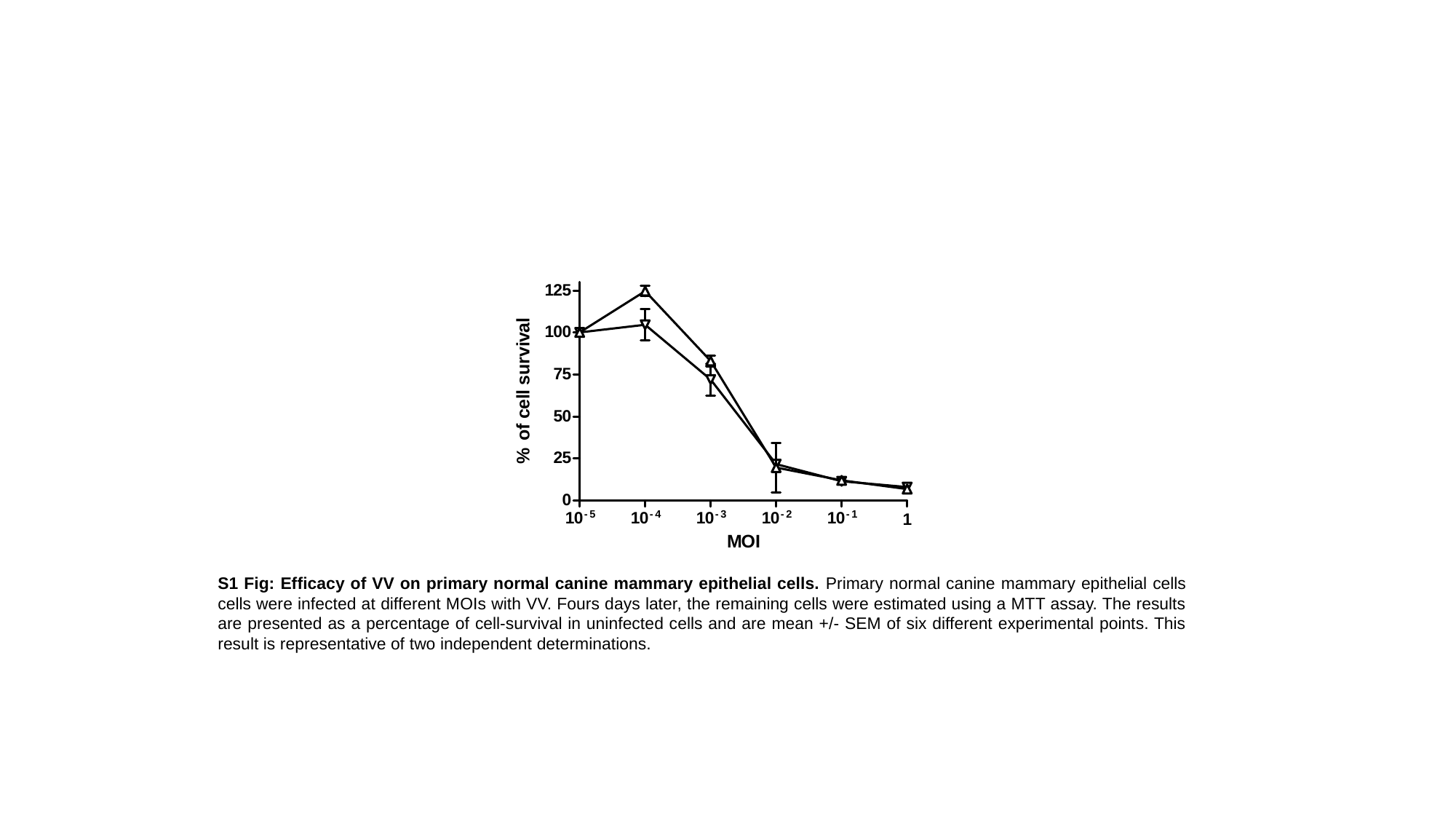

S1 Fig: Efficacy of VV on primary normal canine mammary epithelial cells. Primary normal canine mammary epithelial cells cells were infected at different MOIs with VV. Fours days later, the remaining cells were estimated using a MTT assay. The results are presented as a percentage of cell-survival in uninfected cells and are mean +/- SEM of six different experimental points. This result is representative of two independent determinations.
