# Supplementary material for: Identification of oncolytic vaccinia restriction factors in canine high-grade mammary tumor cells using single-cell transcriptomics
Source: PLoS Pathog. 2020 Oct 19;16(10):e1008660. doi: 10.1371/journal.ppat.1008660 (PMC7595618; doi:10.1371/journal.ppat.1008660)
Supplement: S2 Fig — Non-TNBC (A, B) or TNBC (C, D) cells were infected with a vaccinia virus-Copenhagen strain recombinant in which GFP expression is driven by an immediate-early vaccinia virus promoter (MOI = 5). Three hours after infection, the cells were fixed and stained with propidium iodide (PI). A and B: GFP staining; C and D: propidium iodide staining; M: mininuclei. F, G, H, I.Non-TNBC (white bars) or TNBC (black bars) cells were infected with a vaccinia virus-Copenhagen strain recombinant in which GFP expression is driven by an immediate-early vaccinia virus promoter (MOI = 5). Three hours after infection, the cells were fixed and stained with propidium iodide (PI). The number of PI and GFP positive cells was determined. The percentage of GFP+ cells (F), the mean GFP fluorescence per cell (G), the percentage of mini-nuclei in GFP+ cells (H) and the number of mini-nuclei in nuclei-positive cells (I) are presented. (*** p < 0.001; ** p < 0.01; * p < 0.05; n.s: p > 0.05). The data were obtained from the analysis of 120 images obtained from 2 non-TNBC and 2 TNBC from 4 primary canine specimen. The detail of the samples used to obtain these data is listed in S5 Table. (PPTX) [file ppat.1008660.s002.pptx]

## Slide 1
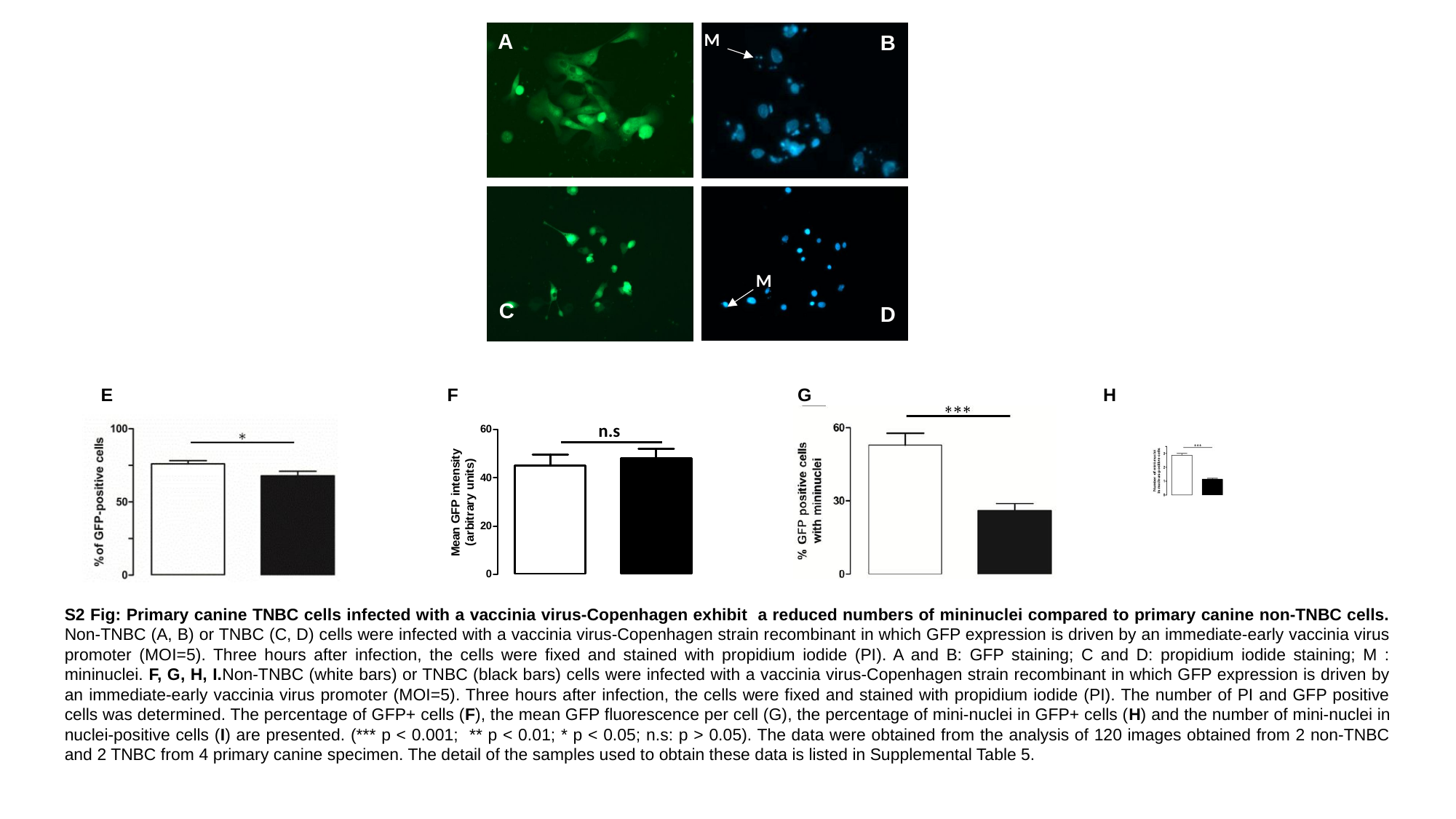

A
M
M
B
C
D
E
F
G
H
n.s
S2 Fig: Primary canine TNBC cells infected with a vaccinia virus-Copenhagen exhibit a reduced numbers of mininuclei compared to primary canine non-TNBC cells. Non-TNBC (A, B) or TNBC (C, D) cells were infected with a vaccinia virus-Copenhagen strain recombinant in which GFP expression is driven by an immediate-early vaccinia virus promoter (MOI=5). Three hours after infection, the cells were fixed and stained with propidium iodide (PI). A and B: GFP staining; C and D: propidium iodide staining; M : mininuclei. F, G, H, I.Non-TNBC (white bars) or TNBC (black bars) cells were infected with a vaccinia virus-Copenhagen strain recombinant in which GFP expression is driven by an immediate-early vaccinia virus promoter (MOI=5). Three hours after infection, the cells were fixed and stained with propidium iodide (PI). The number of PI and GFP positive cells was determined. The percentage of GFP+ cells (F), the mean GFP fluorescence per cell (G), the percentage of mini-nuclei in GFP+ cells (H) and the number of mini-nuclei in nuclei-positive cells (I) are presented. (*** p < 0.001; ** p < 0.01; * p < 0.05; n.s: p > 0.05). The data were obtained from the analysis of 120 images obtained from 2 non-TNBC and 2 TNBC from 4 primary canine specimen. The detail of the samples used to obtain these data is listed in Supplemental Table 5.
