# Supplementary material for: Identification of oncolytic vaccinia restriction factors in canine high-grade mammary tumor cells using single-cell transcriptomics
Source: PLoS Pathog. 2020 Oct 19;16(10):e1008660. doi: 10.1371/journal.ppat.1008660 (PMC7595618; doi:10.1371/journal.ppat.1008660)
Supplement: S3 Fig — Non-TNBC (white bars) or TNBC (black bars) cells were infected at MOI of 0.1 and total RNA were extracted 2, 4 and 8 hours post-infection. The levels of expression of E9L and A27L were determined by quantitative RT-PCR and normalized to the expression of beta-actin. (PPTX) [file ppat.1008660.s003.pptx]

## Slide 1
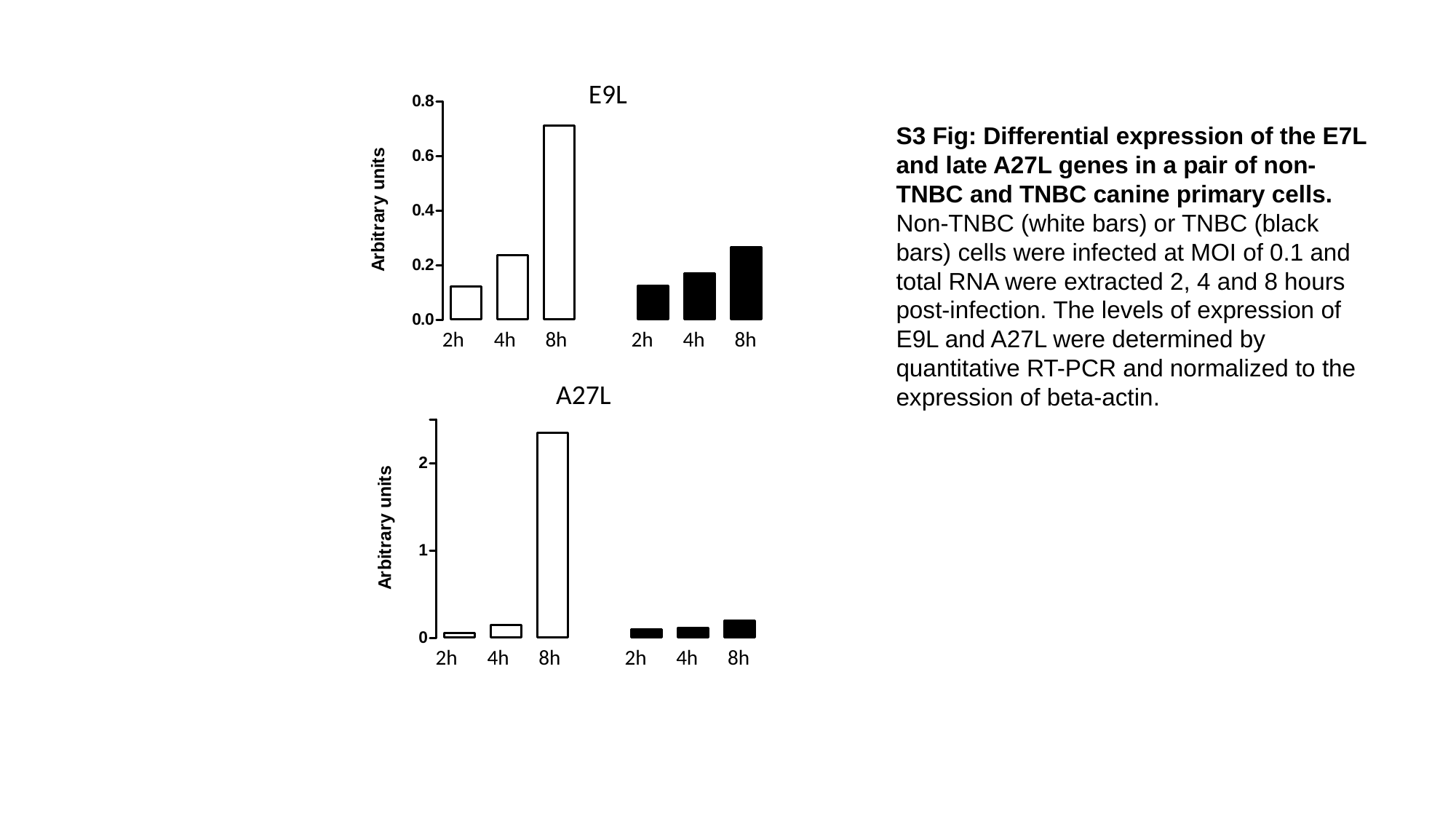

E9L
S3 Fig: Differential expression of the E7L and late A27L genes in a pair of non-TNBC and TNBC canine primary cells. Non-TNBC (white bars) or TNBC (black bars) cells were infected at MOI of 0.1 and total RNA were extracted 2, 4 and 8 hours post-infection. The levels of expression of E9L and A27L were determined by quantitative RT-PCR and normalized to the expression of beta-actin.
2h
4h
8h
2h
4h
8h
A27L
2h
4h
8h
2h
4h
8h
