# Supplementary material for: Identification of oncolytic vaccinia restriction factors in canine high-grade mammary tumor cells using single-cell transcriptomics
Source: PLoS Pathog. 2020 Oct 19;16(10):e1008660. doi: 10.1371/journal.ppat.1008660 (PMC7595618; doi:10.1371/journal.ppat.1008660)
Supplement: S4 Fig — The genes caracteristic of the clusters COL1A2 in the two experiments were anaylsed using IPA. In both cases the transcriptomic signature is associated with TGF-b1 and the top upstream regulator. A: Experiment 1: 69 out of 99 genes consistent with an activation of TGF-b1 (z score 7,037). B: Experiment 2: 77 out of 102 genes have measurements consistent with an activation of TGF-b1 (z score 7,933). (PPTX) [file ppat.1008660.s004.pptx]

## Slide 1
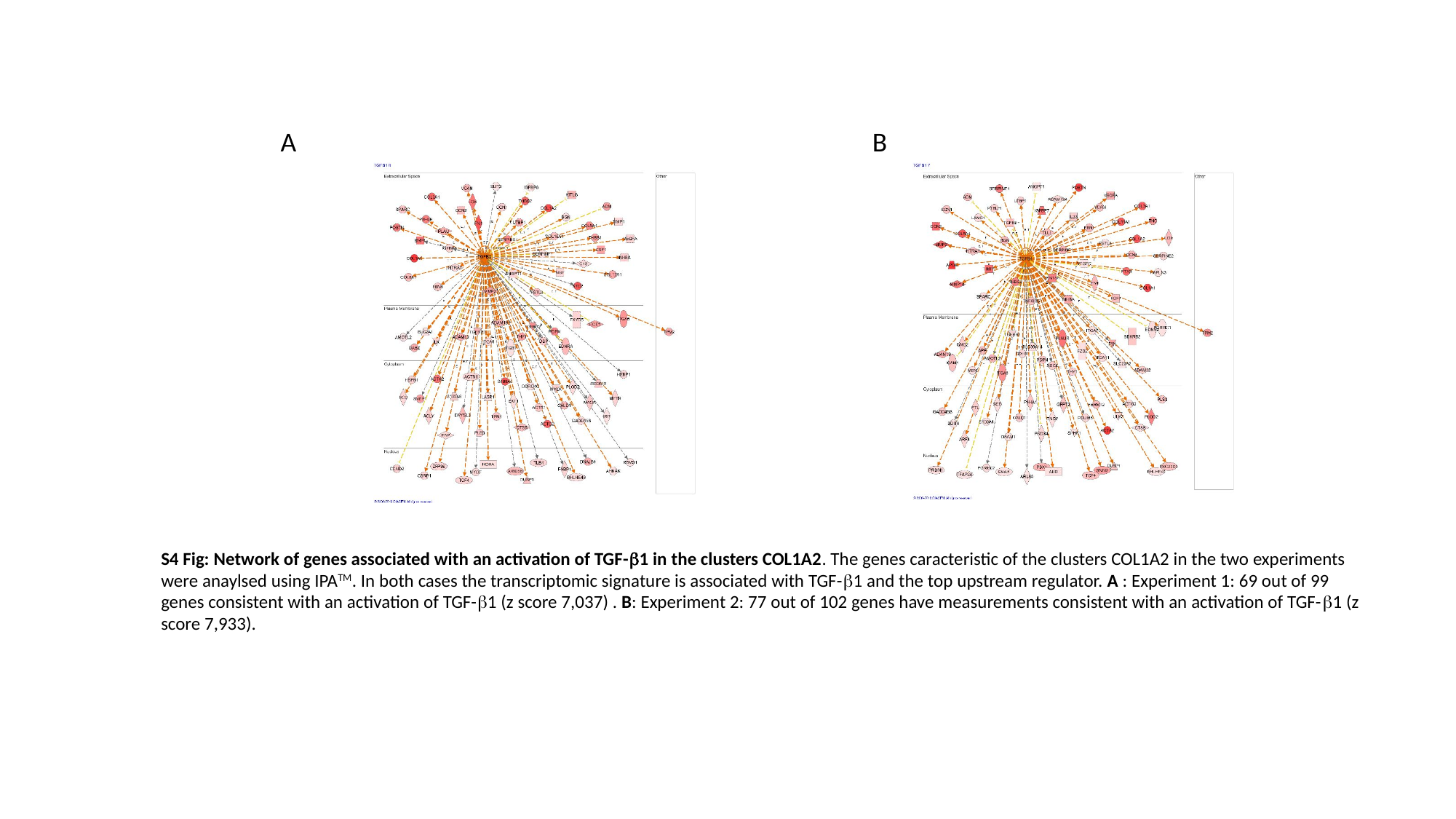

A
B
S4 Fig: Network of genes associated with an activation of TGF-b1 in the clusters COL1A2. The genes caracteristic of the clusters COL1A2 in the two experiments were anaylsed using IPATM. In both cases the transcriptomic signature is associated with TGF-b1 and the top upstream regulator. A : Experiment 1: 69 out of 99 genes consistent with an activation of TGF-b1 (z score 7,037) . B: Experiment 2: 77 out of 102 genes have measurements consistent with an activation of TGF-b1 (z score 7,933).
