# Supplementary material for: Identification of oncolytic vaccinia restriction factors in canine high-grade mammary tumor cells using single-cell transcriptomics
Source: PLoS Pathog. 2020 Oct 19;16(10):e1008660. doi: 10.1371/journal.ppat.1008660 (PMC7595618; doi:10.1371/journal.ppat.1008660)
Supplement: S5 Fig — Primary canine cells from TNBC origin used in single-cell transcriptomics experiments 1 and 2 were infected at different MOIs with VV. Fours days later, the remaining cells were estimated using a MTT assay. The results are presented as a percentage of cell-survival in uninfected cells and are mean +/- SEM of six different experimental points. This result is representative of two independent determinations. (PPTX) [file ppat.1008660.s005.pptx]

## Slide 1
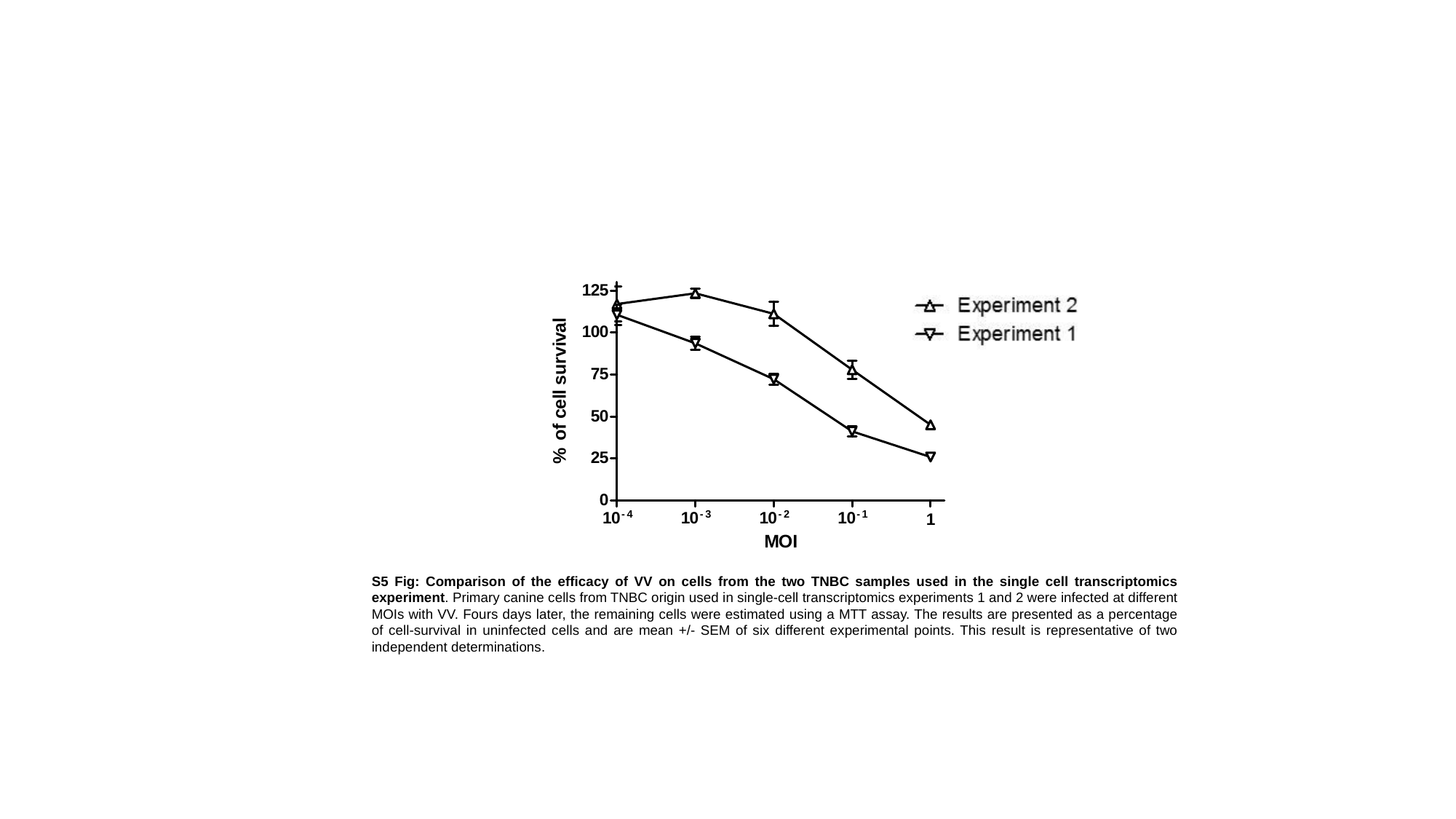

S5 Fig: Comparison of the efficacy of VV on cells from the two TNBC samples used in the single cell transcriptomics experiment. Primary canine cells from TNBC origin used in single-cell transcriptomics experiments 1 and 2 were infected at different MOIs with VV. Fours days later, the remaining cells were estimated using a MTT assay. The results are presented as a percentage of cell-survival in uninfected cells and are mean +/- SEM of six different experimental points. This result is representative of two independent determinations.
