# Supplementary material for: Identification of oncolytic vaccinia restriction factors in canine high-grade mammary tumor cells using single-cell transcriptomics
Source: PLoS Pathog. 2020 Oct 19;16(10):e1008660. doi: 10.1371/journal.ppat.1008660 (PMC7595618; doi:10.1371/journal.ppat.1008660)
Supplement: S1 Table — S1A Table: Differential expression of genes in bystander versus naïve cells (Experiment 1). S1B Table: Differential expression of genes in infected versus bystander cells (Experiment 1). (DOCX) [file ppat.1008660.s006.docx]

| **Specimen** | **Histology** | **Utilization in Figure** |
| --- | --- | --- |
| 16 | normal | Suppl. Fig. 1 |
| 20 | normal | Suppl. Fig. 1 |
| 14 | Benign tumor | Fig. 1C |
| 15 | Benign tumor | Fig. 1C |
| 22 | Benign tumor | Fig. 1C |
| 1 | Carcinoma in situ | Fig. 1C, 1D |
| 3 | Carcinoma in situ | Fig. 1C, 1D |
| 9 | Carcinoma in situ | Fig. 1C, 1D |
| 23 | Carcinoma in situ | Fig. 1C, 1D |
| 5 | Invasive grade 1 | Fig. 1C, D, E; Suppl. Fig. 2 |
| 7 | Invasive grade 2 | Fig. 1A, C, D, E, F; Suppl. Fig. 2 |
| 10 | Invasive grade 2 | Fig. 1C, D; Suppl. Fig. 3 |
| 11 | TNBC-invasive grade 3 | Fig. 1A, C, D, E; Suppl. Fig. 2 |
| 12 | TNBC-invasive grade 3 | Fig. 1C, D |
| 13 | TNBC-invasive grade 3 | Fig. 1C, D; Fig. 2; Fig.3; Fig.4; Suppl. Fig. 5 |
| 19 | TNBC-invasive grade 3 | Fig. 1C, D; |
| 24 | TNBC-invasive grade 3 | Fig. 1C, D, E, F; Fig. 2; Fig.3; Fig.4; Suppl. Fig. 2; Suppl. Fig. 3; Suppl. Fig. 5 |
| 25 | TNBC-invasive grade 3 | Fig. 1C |

**S1 Table: Histological types and utilization of the cells from the different specimen in the figures of the manuscript.**
