# Supplementary material for: Identification of oncolytic vaccinia restriction factors in canine high-grade mammary tumor cells using single-cell transcriptomics
Source: PLoS Pathog. 2020 Oct 19;16(10):e1008660. doi: 10.1371/journal.ppat.1008660 (PMC7595618; doi:10.1371/journal.ppat.1008660)
Supplement: S5 Table — (DOCX) [file ppat.1008660.s010.docx]

| **Specimen** | **Breed** | **Age (year)** | **Type of tissue** |
| --- | --- | --- | --- |
| 1 | Yorkshire Terrier | 12 | Tumor |
| 3 | Fox Terrier cross | 12 | Tumor |
| 5 | Griffon cross | 9 | Tumor |
| 7 | Poodle | 11 | Tumor |
| 9 | Poodle | 11 | Tumor |
| 10 | Fox Terrier | 13 | Tumor |
| 11 | French Bulldog | 8 | Tumor |
| 12 | Bichon frise | 9 | Tumor |
| 13 | Cocker Spaniel | 13 | Tumor |
| 14 | Shih-Tzu | 7 | Tumor |
| 15 | Shih-Tzu | 11.7 | Tumor |
| 16 | Breton Spaniel | 11 | Normal |
| 19 | Mixed breed | 12 | Tumor |
| 20 | Shepherd cross | 12 | Normal |
| 22 | Yorkshire Terrier | 12 | Tumor |
| 23 | Maltese | 10 | Tumor |
| 24 | Cocker Spaniel | 12 | Tumor |
| 25 | Spaniel cross | 13.7 | Tumor |

**S5 Table: Breed, age and type of tissue used to extract low passage, primary cells.**
