# Supplementary material for: Identification of oncolytic vaccinia restriction factors in canine high-grade mammary tumor cells using single-cell transcriptomics
Source: PLoS Pathog. 2020 Oct 19;16(10):e1008660. doi: 10.1371/journal.ppat.1008660 (PMC7595618; doi:10.1371/journal.ppat.1008660)
Supplement: S6 Table — (DOCX) [file ppat.1008660.s011.docx]

| **Specimen** | **Histology** | **Status** | | |
| --- | --- | --- | --- | --- |
|  |  | **ER** | **PR** | **HER2** |
| 16 | Normal tissue | + | + | + |
| 9 | CIS | - | - | + |
| 11 | Grade 3 | - | - | - |
| 12 | Grade 3 | - | - | - |
| 13 | Grade 3 | - | - | - |
| 19 | Grade 3 | - | - | - |
| 24 | Grade 3 | - | - | - |
| 25 | Grade 3 | - | - | - |

**S6 Table: Estrogen receptor (ER), progesterone receptor (ER) and human epidermal growth factor receptor 2 (HER2) status of relevant biopsies used in this study.**
